# Pretargeting for imaging and therapy in oncological nuclear medicine

**DOI:** 10.1186/s41181-017-0026-8

**Published:** 2017-06-06

**Authors:** Clément Bailly, Caroline Bodet-Milin, Caroline Rousseau, Alain Faivre-Chauvet, Françoise Kraeber-Bodéré, Jacques Barbet

**Affiliations:** 1 Service de Médecine Nucléaire, CHU de Nantes, Nantes, France; 2 Service de Médecine Nucléaire, Institut de Cancérologie de l’Ouest, Saint-Herblain, France; 3 Centre de Recherche en Cancérologie Nantes/Angers (CRCNA), Nantes, France; 4 6299 CNRS, Nantes, France; 5 UMR892 Inserm, Nantes, France; 6 Université de Nantes, Nantes, France; 7 GIP Arronax, 1, rue Arronax, 44187 Saint-Herblain cedex, France

**Keywords:** Pretargeting, Immunoscintigraphy, ImmunoPET, Radioimmunotherapy, Bispecific antibody, Avidin-biotin, Oligonucleotides, Click chemistry

## Abstract

**Background:**

Oncological pretargeting has been implemented and tested in several different ways in preclinical models and clinical trials over more than 30 years. Despite highly promising results, pretargeting has not achieved market approval even though it could be considered the ultimate theranostic, combining PET imaging with short-lived positron emitters and therapy with radionuclides emitting beta or alpha particles.

**Results:**

We have reviewed the pretargeting approaches proposed over the years, discussing their suitability for imaging, particularly PET imaging, and therapy, as well as their limitations. The reviewed pretargeting modalities are the avidin-biotin system, bispecific anti-tumour x anti-hapten antibodies and bivalent haptens, antibody-oligonucleotide conjugates and radiolabelled complementary oligonucleotides, and approaches using click chemistry. Finally, we discuss recent developments, such as the use of small binding proteins for pretargeting that may offer new perspectives to cancer pretargeting.

**Conclusions:**

While pretargeting has shown promise and demonstrated preclinical and clinical proof of principle, full-scale clinical development programs are needed to translate pretargeting into a clinical reality that could ideally fit into current theranostic and precision medicine perspectives.

## Introduction

Antibodies have been used as radionuclide carriers for imaging and therapywell before the discovery of monoclonal antibodies. The ability to produce unlimited amounts of homogenous products has prompted the development of antibodies for in vivo imaging and therapy, mostly in oncology. Contrary to the commercial and clinical success of naked therapeutic antibodies, only one labelled therapeutic antibody, Zevalin, for the treatment of B-cell lymphomas (Grillo-López 2002), and a few labelled antibodies for imaging (Goldenberg 1997) remain on the market. There are multiple reasons why labelled antibodies have not been commercially successful. Firstly, the pharmaceutical industry favours non-labelled products, as evidenced by the number of antibody-drug conjugates under development (Thomas et al. 2016), even though they do not outperform labelled antibodies in terms of efficacy or tolerance (Chatal et al. 2016). Secondly, referring physicians are reluctant to prescribe products they do not control.And finally, current therapeutic radiolabelled antibodies have a low therapeutic index..Indeed, radiolabelled antibodies in the form of intact IgG molecules, by far the most commonly used so far, have a very long persistence in the circulation, which can cause severe haematological side-effects. If tumour accretion may reach high levels (more than 10% of injected activity per gram of tumour in mice and as much as 0.1% per gram of tumour in humans), this is only hours or days after activity injection and circulating activities remain elevated for days (Jain et al. 2007).

This explains why, in parallel to the development of therapeutic antibodies and labelled antibodies for tumour imaging, research on alternatives to intact IgG’s has been very active. Antibody fragments and recombinant proteins, such as recombinant single chain Fv fragments, have been produced and tested. Whilst their blood clearance is faster, tumour accretion is reduced and kidney uptake is increased to levels inappropriate for therapy (Jain et al. 2007). Another alternative approach is pretargeting, whereby tumour target antigen binding specificity is obtained by injecting unlabelled antibody derivatives followed by a radiolabelled low molecular weight compound that specifically binds to the tumour and is rapidly cleared from the circulation (Goodwin et al. 1986). The goal is to achieve the targeting performances of the best peptides, such as somatostatin analogues, which have shown their efficacy in neuroendocrine tumours, both for imaging and for therapy (van Essen et al. 2014). Recent results with other low molecular weight compounds such as PSMA or CXCR4 inhibitors (Herrmann et al. 2016; Lütje et al. 2015; Vag et al. 2016) labelled with gallium-68 or fluorine-18 for PET imaging or lutetium-177 for therapy, confirm that a low molecular weight and a fast clearance are important for efficient targeting and reduction of side effects. Nevertheless, because antibodies target a much wider range of antigens with potentially better specificity, they remain of high interest.

Goodwin et al. (Goodwin et al. 1986) and Halpern et al. (Halpern & Dillman 1987) were the first to propose the use of a specific immunoconjugate recognizing both the target antigen and a low-molecular-weight substance carrying the radionuclide to detect or treat tumours. The unlabelled tumour-specific agent and the substance carrying the radionuclide were injected sequentially, with the radioactive compound being administered only after the antibody had localized in the target lesion and its excess was cleared, at least in part, from the circulation. This was first achieved using a bispecific antibody binding both a target antigen and a radiometal-chelate complex (Lollo et al. 1994; Stickney et al. 1991). Since then, many pretargeting studies have shown encouraging preclinical and clinical proof of concept results for both imaging and therapy (Goldenberg et al. 2006). Our historical review highlights these studies. However, after over thirty years of active research, no product has even come near to market approval. The possible reasons for this failure are also considered.

## Review

### Early developments and the rationale of pretargeting

**Fig. 1:**
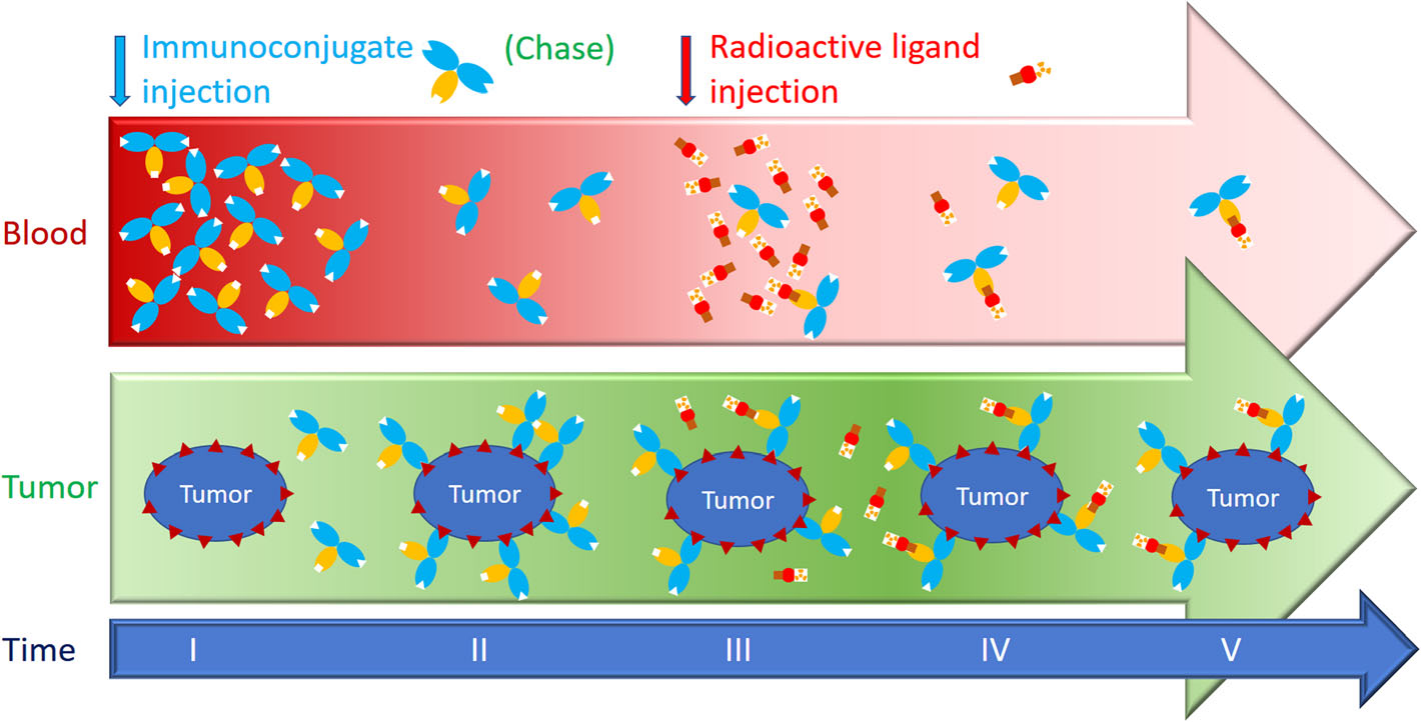
Pretargeting dosing schedule. The unlabelled immunoconjugate is injected first (I). It is allowed to distribute and bind the tumour for several hours or days (II). Then, the radioactive ligand is injected (III). It distributes rapidly and binds the tumour-associated immunoconjugate (IV). It also binds the circulating immunoconjugate that remains in the circulation. The immunoconjugate and the radiolabelled ligand must be carefully designed and the dosing schedule must be optimized to achieve the best tumour to tissue contrast ratios or the best irradiation dose ratio (V). If the amount of immunoconjugate remaining in the circulation is too high, the radiolabelled ligand is trapped in the circulation, reducing the contrast ratios and increasing the irradiation of normal tissues. If the immunoconjugate dose is insufficient, or if the radiolabelled ligand is injected too late, clearance of the immunoconjugate results in its wash-out from tumours and consequently the uptake of radioactivity in the tumour is reduced. To solve the problem, some pretargeting strategies use a clearing agent to chase the excess circulating immunoconjugate (Forero et al. 2004; Houghton et al. 2017; Knox et al. 2000; Paganelli et al. 2001). In other strategies, bivalent haptens, which bind more tightly to cell-bound than to circulating immunoconjugates, are used to carry the radionuclide (Bodet-Milin et al. 2016; Chatal et al. 2006; Gautherot et al. 2000; Kraeber-Bodéré et al. 1999; Kraeber-Bodéré et al. 2006; Kraeber-Bodéré et al. 2015; Peltier et al. 1993; Salaun et al. 2012; Schoffelen et al. 2010; Schoffelen et al. 2013; Schoffelen et al. 2014)

In the early days of pretargeting, the main objective was tumour imaging. Indium-111 was the radionuclide of choice, and several pretargeting approaches were proposed (Goldenberg et al. 2006). Generally, antibodies or antibody fragments were used to recognize and bind the tumour cell target antigen, and a second agent was needed to bind the small molecule that carried the radioactive payload. Figure 1 schematizes a typical dosing schedule. Originally, the indium-benzyl-EDTA complex was used as a hapten, and bispecific antibodies binding carcinoembryonic antigen (CEA) and the hapten were used for imaging tumours (Lollo et al. 1994; Stickney et al. 1991). Biotin, which binds very tightly to avidin or streptavidin, was also proposed (Hnatowich et al. 1987; Pimm et al. 1988). The use of antibodies conjugated to an oligonucleotide binding to a radiolabelled complementary oligonucleotide was also proposed a few years later (Bos et al. 1994; Liu et al. 2002). Table 1 summarizes some of these approaches.

**Table 1:** Examples of pretargeting methods and applications

Pretargeting agent	Target antigen	Chase	Radioactivity vector	Pretargeted radionuclides	Results	References
Murine antibody or ScFv-streptavidin conjugate	Ep-CAM	biotinylated galactosyl-human serum albumin	DOTA-biotin	Yttrium-90	Clinical radioimmunotherapy of lung cancer and lymphoma	(Hnatowich et al. 1987; Houghton et al. 2017)
					Pros: targeting efficacy, high tumour/non-tumour ratio	
					Cons: complexity (3 compounds), presence of endogenous biotin, toxicity (lung cancer), immunogenicity of streptavidin	
Murine biotinylated antibody	CEA, tenascin	Avidin + streptavidin	DOTA-Biotin	Yttrium-90	Clinical radioimmunotherapy of brain tumours	(Heskamp et al. 2017)
					Pros: targeting efficacy, high tumour/non-tumour ratio	
					Cons: complexity (4 compounds), immunogenicity of avidin/streptavidin	
Murine and chimeric bispecific antibody	CEA	None	Indium-EDTA haptens	Indium-111	Clinical immunoscintigraphy	(Goldenberg 1997)
					Pros: high tumour/non-tumour ratio, tumour imaging in the liver	
					Cons: low tumour uptake, moderate sensitivity	
Murine and chimeric bispecific antibody (chemically conjugated Fab)	CEA	None	Bivalent haptens	Indium-111	Clinical immunoscintigraphy, radioimmunotherapy	(Le Doussal et al. 1993; Lütje et al. 2015; McBride et al. 2009; Paganelli et al. 2001)
				Iodine-131	Pros: targeting efficacy, high tumour/non-tumour ratio, evidence of therapeutic effect in the clinic	
					Cons: difficulties in the production of bispecific antibodies	
Humanized bispecific antibody (Dock and Lock)	CEA, CD20, Trop2	None	Bivalent haptens	Gallium-68	Clinical immunoscintigraphy, radioimmunotherapy and immuno-PET, preclinical alpha-radioimmunotherapy	(Schoffelen et al. 2010; Schoffelen et al. 2013; Schoffelen et al. 2014; Sharkey et al. 2003; Sharkey et al. 2005)
				Lutetium-177	Pros: high tumour/non-tumour contrast ratio in PET imaging	
				Bismuth-213	Cons: Insufficient tumour irradiation for lutetium-177 therapy	
Murine antibody-oligonucleotide conjugate	Carcinoembryonic antigen	None	Complementary Morpholino oligonucleotide	Technetium-99 m	Preclinical targeting studies	(Halpern & Dillman 1987)
					Pros: good tumour/non-tumour contrast ratio	
					Cons: preparation of antibody-oligonucleotide conjugates	
Affibody-oligonucleotide conjugate	HER2	None	DOTA-peptide nucleic acid	Indium-111	Preclinical targeting and imaging studies	(Yao et al. 2004)
					Pros: very good tumour/non-tumour contrast ratio	
					Cons: preparation of antibody-oligonucleotide conjugates	
Humanized antibody-trans-cyclo-octene conjugate	TAG72, GPA33, CA19.9	None or tetrazine-conjugated albumin attached to galactose or polystyrene beads	DOTA-PEG_7_-tetrazine	Indium-111, copper-64, lutetium-177, zirconium-89	Preclinical targeting; immunoscintigraphy, PET imaging and dosimetry studies	(van Duijnhoven et al. 2015; van Essen et al. 2014; van Schaijk et al. 2005)
					Pros: good tumour uptake and tumour/non-tumour contrast ratios, easy preparation of the reagents	
					Cons: need for a chase step to achieve excellent results	
Diabody- or Affibody-trans-cyclo-octene conjugate	TAG72, HER2	None	DOTA-PEG_10_-tetrazine	Lutetium-177	Preclinical targeting and imaging studies	(Vugts et al. 2013; Yao et al. 1995)
					Pros: good tumour uptake and tumour/non-tumour contrast ratios, easy preparation of the reagents, no need for a chase step	
					Cons: possible problem of kidney uptake for therapy	

Whilst results were encouraging, low tumour uptake was observed if the injected dose of bispecific antibody was low or the delay between bispecific antibody and hapten injections was too long. Conversely, with a high dose of bispecific antibody and a short delay, tumour uptake was appreciable, but retention of the radioactive hapten in the circulation was high and protracted. Similar problems were observed using the avidin-biotin approach.There was clearly a need for significant improvements.

### Pretargeting with the avidin-biotin system

With the avidin-biotin system, a chase step was proposed to remove a large fraction of the circulating antibody conjugate and subsequently reduce the background activity in normal tissues (Yao et al. 1995). However, by this time, the emphasis had shifted from tumour imaging to cancer therapy. Indeed, tumour imaging using antibodies is highly specific for target antigen expression and tumours not expressing the target antigen score as false-negatives in tumour detection. In addition, positron emission tomography (PET) with ^18^F-fluoro-deoxy-glucose (FDG) had appeared as a general, sensitive, and sufficiently specific tumour detection method: immunoscintigraphy and pretargeted immunoscintigraphy appeared obsolete. However, pretargeting had been shown to be capable of delivering radiation doses to tumours, thus shifting the interest from tumour detection to tumour therapy.

Clinical studies were then performed using complex protocols involving three to five injections. Very good responses were obtained in patients with glioblastomas or anaplasic gliomas by Paganelli and co-workers using the sequential injection of (i) a biotinylated antibody (recognizing tenascin), (ii) avidin, to clear excess biotinylated antibody, (iii) streptavidin to decorate the tumour cell bound biotinylated antibody and (iv) yttrium-90-labelled DOTA-biotin (Paganelli et al. 2001). Twelve of the 48 treated patients benefited from > 25% tumour regression, and 8 of the 48 patients had a tumour response lasting for more than 12 months. The avidin-biotin system was also used for pretargeted radioimmunotherapy of lung cancer, but the lack of specificity of the targeting antibody resulted in severe toxicity (Knox et al. 2000). Pretargeting with the avidin-biotin system also showed promise in the treatment of lymphoma in both preclinical and clinical trials (Axworthy et al. 2000; Forero et al. 2004).

The advantage of the avidin-biotin system is that antibody-streptavidin or antibody-biotin conjugates are easily developed. In addition, available biotin derivatives may be readily radiolabelled. Although the published results are impressive, problems related to the immunogenicity of avidin and streptavidin have not been solved.

### Pretargeting with bispecific antibodies and radiolabelled haptens: The Affinity Enhancement System

Bispecific antibodies may be prepared by a variety of techniques. In the early days, chemical conjugates of antibody fragments were used (Stickney et al. 1991) whereas hybrid hybridomas and quadromas were also considered (Kranenborg et al. 1995; van Schaijk et al. 2005). The presence of excess bispecific antibody in the circulation at the time of radiolabelled hapten injection was a problem, and resulted in relatively slow activity clearance and non-specific deposition in normal tissues. Increasing the time delay between the two injections resulted in poor tumour activity uptake. To circumvent this, Le Doussal and co-workers proposed the Affinity Enhancement System or AES (Le Doussal et al. 1990). This used a bivalent hapten that could cross-link bispecific antibody molecules at the surface of target cells and bind with enhanced affinity (or avidity), whereas, in the circulation, binding remained rapidly reversible, avoiding the need for a chaseof excess bispecific antibody. The interest of using bivalent haptens was independently confirmed by Goodwin and co-workers (Goodwin et al. 1992; Goodwin et al. 1994), who also proposed pretargeting for therapy using a monoclonal antibody raised against the yttrium-DOTA hapten, and later by Boerman and co-workers (Boerman et al. 1999), with a hybrid hybridoma of the anti-renal cell carcinoma antibody G250 and an anti-DTPA-indium antibody.

In the original studies performed by our group in association with the French company Immunotech (Marseille, France), the bispecific antibody was prepared by chemical conjugation of the Fab fragment of the anti-CEA antibody F6 to the Fab fragment of an antibody recognizing the indium-DTPA complex. The bivalent hapten was prepared by reacting tyrosyl-lysine with DTPA anhydride. Surprisingly, the distance between the two indium-DTPA moieties may be quite short and the tyrosyl-lysine dipeptide, substituted by DTPA on both the α-NH_2_ of tyrosine and the ε-NH_2_ of lysine, allows for the simultaneous *in vitro* binding of two anti-DTPA-indium antibodies (Le Doussal et al. 1990). This bivalent hapten could be labelled with indium-111, but also radio-iodinated. However, the antibody specificity for the indium-DTPA complex limited radiolabelling to indium-111 or radioactive iodine. Bispecific antibodies recognizing another hapten, the histidine-succinyl-glycine (HSG) pseudo-peptide, were prepared and tested successfully with bivalent HSG haptens labelled with a variety of radionuclides (Janevik-Ivanovska et al. 1997).

### Pretargeted radioimmunodetection and pretargeted radioimmunotherapy (RIT) with bispecific antibodies and bivalent haptens (AES)

Clinical AES pretargeted imaging using indium-111 scintigraphy produced high contrast images (Le Doussal et al. 1993; Peltier et al. 1993), particularly in medullary thyroid carcinoma (MTC), which consistently expresses CEA. However, successful tumour imaging required a long delay (2 to 4 days) between the bispecific antibody injection and injection of the labelled bivalent hapten, and high contrast images were obtained 24 hours later.The development of FDG-PET imaging further limited the interest of pretargeting even if the specificity was very high.

AES pretargeting could increase the radioimmunotherapy therapeutic index because it increases the tumour-to-normal tissue uptake ratio and increases radiation doses delivered to tumour cells. This was shown in preclinical models (Gautherot et al. 2000; Kraeber-Bodéré et al. 1999). The toxicity, pharmacokinetics, dosimetry and anti-tumour activity of the murine anti-CEA bispecific antibody F6x734 and the bivalent indium-DTPA hapten labelled with iodine-131 were evaluated in a phase I/II clinical trial in 26 patients with recurrent MTC (Kraeber-Bodéré et al. 2006). Haematological toxicity was the dose-limiting toxicity and the maximum tolerated activity was relatively low (1.8 GBq/m^2^). Whilst therapeutic responses were observed in only a small number of patients with small tumour burdens and after repeated courses of pretargeted RIT, long-term disease stabilization in a large number of the MTC patients (53%) was documented by morphological imaging (computed tomography, MRI) and serial calcitonin and CEA measurements. The overall survival of 29 MTC patients treated by pretargeted RITA was compared to 39 contemporaneous untreated patients (Chatal et al. 2006). These patients were stratified according to calcitonin and CEA doubling times. Overall survival (OS) was significantly longer for high-risk treated patients (calcitonin Ct doubling time < 2 years) compared to high-risk untreated patients (median OS, 110 vs. 61 months; *P* < 0.030). Toxicity was mainly haematological, partly because of the frequent diffuse bone marrow tumour involvement. However, after treatment, patients with bone/bone marrow disease had a longer survival than patients without such involvement (10-year OS of 83% vs. 14%; *P* < 0.023). Interestingly, no other toxicity, especially renal toxicity, was reported. These results were confirmed in a prospective multicentric phase II trial. Treatment of progressive MTC patients (calcitonin doubling times shorter than 5 years) achieved a disease control rate (durable stabilization plus objective response) of 76.2% according to RECIST morphological imaging criteria, a durable complete response of more than 40 months in 1 patient (2.4%) and durable stable disease (≥6 months) in 31 patients (73.8%) (Chatal et al. 2006). After RIT, 21 of 37 assessed patients (56.7%) showed a ≥100% increase in calcitonin or CEA doubling times or prolonged decrease of the biomarker concentration. Hematologic toxicity (grade 3 and 4) was observed in 54.7% of the patients and myelodysplastic syndrome reported in 2 cases, including 1 previously heavily treated.

### Pretargeted PET imaging

Recently, antibodies labelled with positron-emitters have been tested for PET imaging. Because of the slow pharmacokinetics of these antibodies, ong half-life positron emitters, such as zirconium-89 (half-life: 78.4 hours), were the first to be tested (Salaun et al. 2012). Antibodies labelled with copper-64 also provide good contrast images, but a shorter time interval is necessary because of the short radionuclide half-life (12.7 hours). Unfortunately the dosimetry is not very favourable, because zirconium-89 emits a high-energy gamma photon and the copper-64 positron branching ratio is low (18%). In addition, the imaging procedure cannot be performed within a single day after activity injection.

**Fig. 2:**
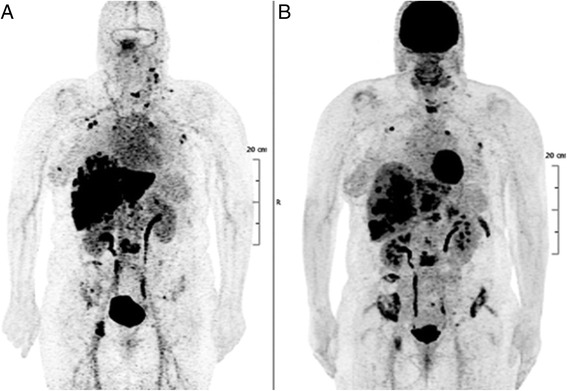
PET imaging of a patient with metastatic breast carcinoma. **a** Pretargeted immuno-PET performed using the TF2 anti-CEA bispecific antibody and the ^68^Ga-labelled IMP-288 peptide detects more lesions than FDG-PET (**b**)

Pretargeting could improve the performance of immuno-PET and allow for the use of short half-life positron-emitting radionuclides such as gallium-68 or fluorine-18 that would reduce patient irradiation. A few years ago Immunomedics Inc. developed the Dock-and-Lock™ (DNL™) technology for producing humanized bispecific antibodies (Vugts et al. 2013). These consist of the regulatory subunits of cAMP-dependent protein kinase fused with one antibody Fab fragment, and the anchoring domains of A kinase fused with the other Fab fragment This allows a very efficient production of bispecific trivalent antibodies, with one site binding the hapten and two sites binding the tumour antigen. Several DNL™ conjugates binding CEA (TF2), CD20 (TF4), a mucin antigen expressed by pancreatic tumours (TF10), and the Trop-2 antigen (TF12) have been described (Gold et al. 2008; Rossi et al. 2006; Sharkey et al. 2005). In addition, using the histamine-succinyl-glycine (HSG) pseudo-peptide allowed construction of a new bivalent hapten, IMP288, permitting a variety of radionuclides to be used (Sharkey et al. 2012). Several studies showed that DNL™ bispecific antibodies were particularly well-suited to delivering short half-life radionuclides. In preclinical experiments, high contrast PET images could be obtained within an hour after radiolabelled hapten injection using gallium-68 and fluorine-18 (McBride et al. 2009; Sharkey et al. 2003). Extremely small tumours could be detected in mice. Pretargeted immuno-PET was then studied in the clinic for several cancers known to express CEA: breast cancers (Schoffelen et al. 2010), colorectal carcinoma and medullary thyroid cancers (Kraeber-Bodéré et al. 2015). Preliminary imaging results were very promising in the three tumours as shown for a breast cancer patient in Fig. 2. In all cases, pretargeted immuno-PET was able to detect very small tumour lesions after optimizing the TF2/IMP-288 molar dose ratio and the pretargeted delay for the anti-CEA bispecific antibody TF2 and ^68^Ga-labeled IMP288 bivalent hapten. High tumour uptake was obtained with this approach in patients with relapsed MTC and HER2-negative breast cancer by injection of 120 nmol of TF2 and 6 nmol of ^68^Ga-IMP-288, 30 h later. In some cases, immuno-PET allowed detection of lesions not detected by F-DOPA-PET, considered as the reference for PET imaging in MTC.

### Current research on pretargeting

Based on the excellent PET imaging results using these new pretargeting agents, a clinical optimization studies were conducted to assess the anti-CEA × anti-HSG bispecific antibody TF2 and the radiolabelled hapten-peptide ^177^Lu-IMP288 in patients with metastatic CRC (Schoffelen et al. 2013). Different schedules were studied, and the best tumour targeting was achieved with a 1-day pretargeting interval, a high TF2 dose (150 mg) and a low (25 μg) peptide dose. Rapid and selective tumour uptake was seen within 1 h after the peptide injection, with high tumour-to-tissue ratios at 24 h. High activities of ^177^Lu-labelled IMP288 (2.5-7.4 GBq) were well tolerated, with some manageable reactions during the TF2 infusions and transient thrombocytopenia (grades 3–4) in 10% of the patients. Calculated radiation doses delivered to the kidneys and red bone marrow were relatively low (Schoffelen et al. 2014), allowing repeated administrations. Subsequently, two phase-I clinical trials were performed in France in patients with metastatic CEA-positive lung carcinoma using ^177^Lu-labelled IMP288, and in metastatic CRC patients using fractionated injections of TF2 and ^90^Y-IMP288. The therapeutic results were disappointing because of the relatively fast wash-out of tumour activity that limited tumour absorbed doses, especially with the long half-life lutetium-177.

Preclinical pharmacokinetic and dosimetry studies have also demonstrated that DNL™ bispecific antibodies, because of their relatively fast blood clearance, could efficiently deliver short half-life radionuclides to tumours (Frampas et al. 2011). Short half-life radionuclides and particularly short half-life alpha-particle-emitting radionuclides, such as astatine-211 or bismuth-213, are considered for therapy, using both the avidin-biotin (Yao et al. 2004) and the AES approaches (Heskamp et al. 2017).

### Alternative approaches in pretargeting

In addition to the avidin-biotin and bispecific antibody approaches, two other alternatives have been proposed. The first relies on using the recognition between complementary DNA sequences. An antibody is derivatized with one oligonucleotide and the radioactivity is carried by an oligonucleotide of complementary sequence. This approach was proposed by Bos and co-workers (Bos et al. 1994) in 1994 and has not been very actively explored, despite the progress in the synthesis of oligonucleotides resistant to in vivo degradation. The other more recent approach is based on bio-orthogonal chemistry using the inverse-electron-demand Diels–Alder reaction between a tetrazine and a strained trans-cyclooctene (TCO) derivative. Rossin and co-workers first demonstrated that this reaction was fast enough and specific to be effective in vivo at the very low concentrations involved in cell targeting (Rossin et al. 2010; Rossin et al. 2013). In this seminal paper, a proof of concept of specific tumour uptake was achieved, but the tumour to normal tissue ratios were modest (Rossin et al. 2010): when an intact IgG is used to prepare the pretargeting immunoconjugate, the presence of excess circulating immunoconjugate at the time of injection of the click-chemistry partner reacts with the labelled molecule and reduces its clearance, as observed with the other pretargeting systems many years earlier. Similar biodistribution results were obtained in another system that also showed positive PET imaging (Zeglis et al. 2013). In a recent paper, Hougton et al. evaluated the click chemistry approach for therapy. Whilst the activity accretion in tumours was high the circulating activity was also high and declined slowly. To solve this problem, Rossin and co-workers used a chase step that considerably improved tumour uptake and tumour to non-tumour ratios (Houghton et al. 2017). As with the avidin-biotin system, the use of a chase step could therefore be a solution, but would be rather cumbersome with a risk of immunogenicity and hypersensitivity side-effects.

The click chemistry pretargeting approach has been tested with a fast clearing antibody derivative: a 60 kDa diabody (van Duijnhoven et al. 2015). High tumour uptake (6.9% injected dose/g) and tumour to blood ratios were observed, with reasonable kidney uptake, and without a chase step demonstrating that faster immunconjugate clearance could be a solution. Tolmachev and co-workers went a step further by using an Affibody, a very small binding protein of 7 kDa, as pretargeting agent (Altai et al. 2016). Tumour accretion and clearance of the pretargeting agent were fast enough to establish a large difference between the amount of Affibody bound to the tumour and that remaining in the circulation. The same Affibody was also used with very good pretargeting results using the oligonucleotide (or peptide nucleotide in that case) approach (Honarvar et al. 2016). Further studies will show whether this promising approach can overcome the current limitations of pretargeting.

## Conclusions and perspectives

Pretargeting originated in the mid-80s, and has been implemented in several different ways and tested in preclinical models and clinical trials. Despite highly promising results in preclinical tumour models, as well as in early phase clinical trials, pretargeting has not yet come close to market approval. However, pretargeting remains as potentially the ultimate theranostic, combining PET imaging with short-lived positron emitters and therapy with radionuclides emitting beta or alpha particles. Producing the required humanized recombinant immunoconjugates remains a challenge. Quadromas and chemically-coupled bispecific antibodies are difficult and expensive to prepare under GMP conditions and may not have the required purity for clinical development. DNL^TM^ bispecific antibodies proved very efficient for imaging, but not as effective for therapy. Bispecific diabodies and triabodies that appear very appropriate in terms of molecular weight and stability (Rossi et al. 2005) have not been produced in the necessary mass quantities so far. Adaptation of older approaches that use avidin biotin or bispecific anti-tumour x anti-hapten antibodies with pretargeting using in vivo click chemistry show promise. The use of small binding proteins for pretargeting may also offer a new perspective. Indeed, these binding proteins have fast in vivo kinetics, but tend to deliver very high activity to the kidneys (as do most peptides), when radiolabelled with metal radionuclides. Pretargeting could be a way to overcome the problem and provide a new targeting approach for both imaging and therapy. Pretargeting is also one way to use short-lived radionuclides to image and treat cancers that do not over-express receptors or enzymes that could be targeted with small peptides or inhibitors.

In conclusion, pretargeting remains quite attractive, particularly for PET imaging and therapy, in a theranostic perspective. However, it requires careful optimization, both for the design of the appropriate pretargeting reagents, bispecific immunoconjugates binding tumour antigens and the small molecule selected to carry the activity, and for the definition of dosing and administration schedules. Full-scale clinical development programs remain needed to translate pretargeting into a clinical reality.

## Abbreviations

CEA: Carcinoembryonic antigen
DNL™: Dock-and-Lock™
DTPA: Diethylene triamine pentaacetic acid
FDG: ^18^F-fluoro-deoxy-glucose
F-DOPA: L-3,4-Dihydroxy-6-[^18^F]fluorophenylalanine
HSG: Histidine-succinyl-glycine
MTC: Medullary thyroid carcinoma
OS: Overall survival
PET: Positron emission tomography
RIT: Radioimmunotherapy.
